# High-performance flexible Ag nanowire electrode with low-temperature atomic-layer-deposition fabrication of conductive-bridging ZnO film

**DOI:** 10.1186/s11671-015-0810-x

**Published:** 2015-02-28

**Authors:** Ya-Hui Duan, Yu Duan, Ping Chen, Ye Tao, Yong-Qiang Yang, Yi Zhao

**Affiliations:** State Key Laboratory on Integrated Optoelectronics, College of electronic science and engineering, Jilin University, 2699 Qianjin Street, Jilin, 130012 China

**Keywords:** Ag nanowire, Zinc oxide, Low-temperature ALD process

## Abstract

As material for flexible transparent electrodes for organic photoelectric devices, the silver nanowires (AgNWs) have been widely studied. In this work, we propose a hybrid flexible anode with photopolymer substrate, which is composed of spin-coating-processed AgNW meshes and of zinc oxide (ZnO) prepared by low-temperature (60°C) atomic layer deposition. ZnO effectively fills in the voids of the AgNW mesh electrode, which is thus able to contact to the device all over the active area, to allow for efficient charge extraction/injection. Furthermore, ZnO grown by low temperature mainly relies on hole conduction to make the anode play a better role. Hole-only devices are fabricated to certify the functionality of the low-temperature ZnO film. Finally, we confirm that the ZnO film grown at a low temperature bring a significant contribution to the performance of the modified AgNW anode.

## Background

With the rapid growth of research in flexible electronics, there is enormous demand for both transparent and reflective bendable electrodes [1]. Carbon nanotubes [2], grapheme [3], conductive polymers [4], and random networks of metallic nanowires [5] have been considered as promising flexible transparent conductors. Silver nanowires (AgNWs) have been considered as one of the most promising materials to replace indium tin oxide (ITO) for flexible optoelectronic devices thanks to their excellent electrical, optical, and mechanical properties [6-8]. There are two critical issues that currently exist in AgNW films. First, AgNW films deposited on bare substrates are highly coarse [9]. Second, they could be easily removed by adhesion or friction [6]. These problems have been solved by a peel-off process combined with a flexible polymer substrate [10]. But still there are gaps between nanowires during the process of cross-linking the polymer. This phenomenon poses a problem which needs to be resolved, that of these gaps restricting the injection of carriers, which is not conducive to reaching its highest performance.

Being a wide-band-gap semiconductor (a gap of approximately 3.4 eV at room temperature) with a high thermal and chemical stability, zinc oxide (ZnO) has recently attracted a great scientific interest due to its possible applications in electronics and optoelectronics. Thin films of ZnO can be prepared by several growth techniques, including pulsed laser deposition [11,12] and radio-frequency magnetron sputtering [13,14]. However, the quality of ZnO films obtained by these methods depends on either a high growth temperature (350°C to 450°C) or a post-growth annealing process. Atomic layer deposition (ALD) has emerged as an important technique for a variety of applications based on large area uniformity, precise thickness control, and highly conformal deposition. Most importantly, it can be applied under low-temperature growth, which is crucial for the fabrication of low-cost and flexible electronics components [15-17]. Based on the conformality, continuous, and pinhole-free films achieved by ALD [18], ZnO films can be used for encapsulation [19]. The electrical properties of ZnO are also a major research focus [20]. The conductivity of this material is strongly defect related. The control of defects is very important for related applications. However, the exact value of free-carrier concentration found in ZnO thin films strongly depends on the growth method and the parameters used in the process. Probably, the most important factor that influences the ZnO film electric parameters is the growth temperature, as it controls the thermodynamics of the growth process [21]. High-temperature deposition processes can intensify the oxygen vacancy or Zn interstitial formation and contribute in this way to the high level of n-electron doping [22]. We obtain ZnO films at the extremely low temperature of 60°C and combine the ZnO films with AgNW anodes. In this work, we first apply the ZnO film as an auxiliary layer of the electrode to form AgNW/ZnO hybrid electrode. By the ALD method, a thin, relatively low viscosity of diethylzinc ((DEZn) [Zn(C_2_H_5_)_2_]) is applied, through which H_2_O molecules could easily penetrate into the porous AgNW network to form a conductive-bridging ZnO film and fill up the unoccupied spaces among the nanowires, forming the second dense conductive film alongside the AgNW network after the photopolymer is cured. When applying this approach, ZnO fills the vacancy between nanowires to a sufficient degree, which resolves the problem that the gaps between nanowires restrict the injection of carriers. As a result, the injection of carriers could be effectively improved. This approach is conducive to making mesh electrodes like nanowire electrodes reach a better performance. At the same time, the low temperature at which ZnO is grown simultaneously guarantees the flexibility and the mechanical properties of the plastic substrate, which is suitable for the development of flexible electronic devices.

## Methods

**Figure 1:**
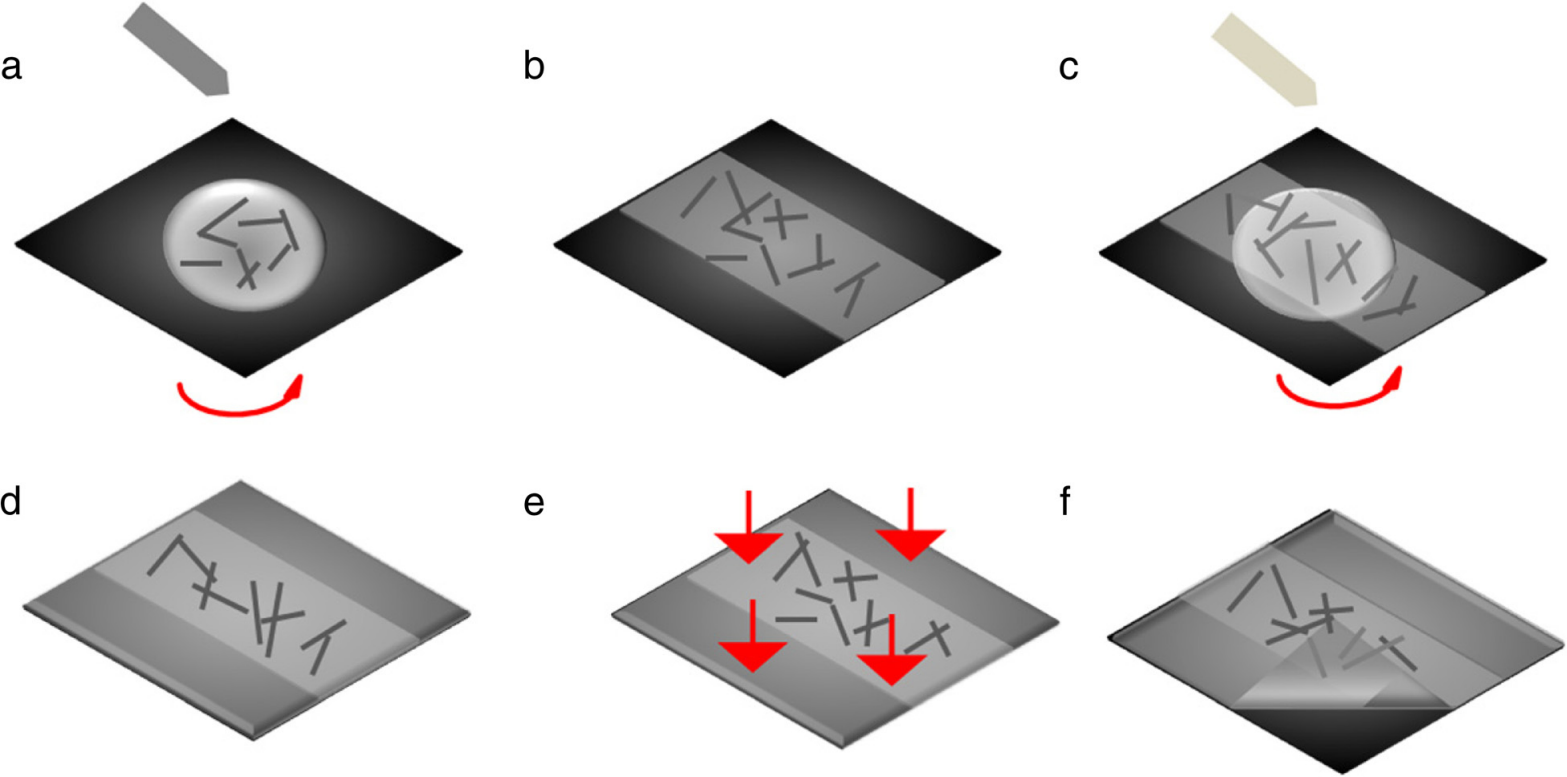
**Ultrasmooth AgNW anode fabricated on photopolymer substrate by spin-coating process and peel-off process. (a)** AgNW suspension drop down on Si substrate and spin-coating at 8,000 rpm for 30 s; **(b)** dry in ambient environment room temperature; **(c)** NOA63 drop down and spin-coating for first spinning at 300 rpm for 15 s, second spinning at 800 rpm for 15 s; **(d)** after spin-coating; **(e)** UV curing at 300 W (370 nm) for 4 min; **(f)** peel-off process.

The AgNW from BlueNano Company (Cornelius, NC, USA) has average dimensions of 90 nm × 25 μm. We adopt an AgNW solution concentration of 5 mg/ml, which we have optimized in our previous work and have shown optimal electrical and optical properties. The fabrication of ultrasmooth AgNW anodes on a photopolymer (NOA63, Norland Optics, Norland Products, Cranbury, NJ, USA) substrate is shown in Figure 1. Initially, an AgNW suspension is shaken for 5 min to form a uniform dispersion and spin coated onto a pre-cleaned Si substrate for 30 s at 8,000 rpm. The AgNW then dries in the ambient environment at room temperature to evaporate residual solvent and form a homogeneous and conductive submicron film of AgNW [23]. NOA63 is then spin coated; the first spinning lasting 15 s at 300 rpm and the second spinning 15 s at 800 rpm. The purpose is to make the glue evenly spread; then, the excess glue is shaken off. The photopolymer film is then next cast on top of the AgNW film, followed by an ultraviolet light treatment at a wavelength of 370 nm and 300 W for 4 min. Finally, in order to peel off AgNW and NOA63 from the Si substrate, we pry an edge of the cured photopolymer from the Si substrate using forceps. This film is easily removed with the AgNW embedded in the photopolymer film [24]. Finally, we obtain the flexible AgNW electrode.

**Figure 2:**
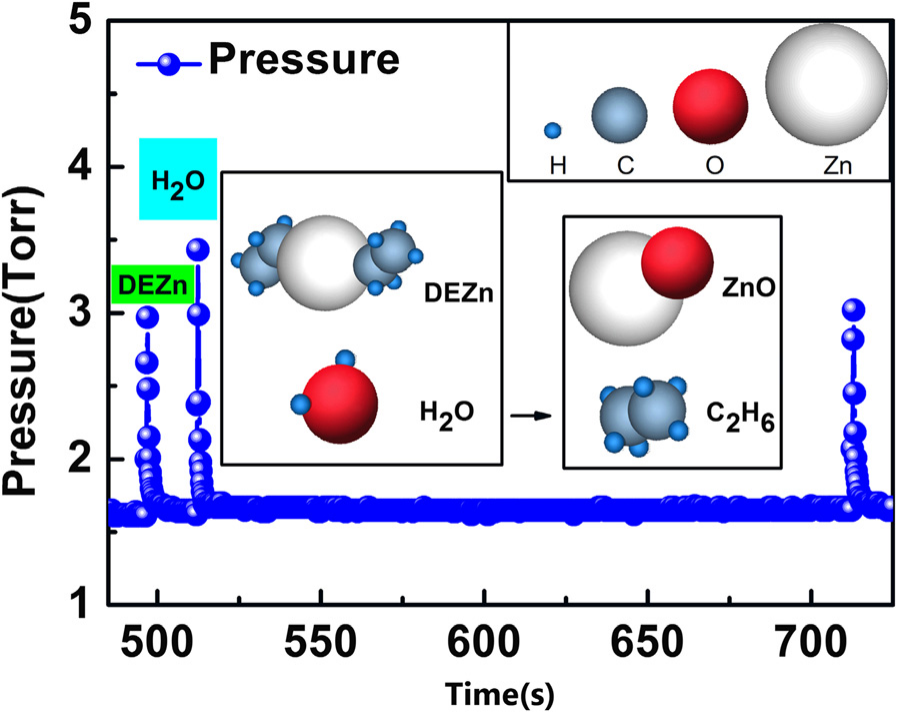
**Precursor pulse of Zn(C**_**2**_ **H**_**5**_ **)**_**2**_ **and H**_**2**_ **O during ZnO film deposition in ALD process.**

We grow the ZnO thin films in the ALD process at an extremely low temperature using the LabNano 9100 ALD system by Ensure Nanotech Inc. (Beijing, China). The chamber pressure is 3 × 10^−2^ Pa. Zn(C_2_H_5_)_2_ and H_2_O are used as the precursors of Zn and O, respectively. The reaction is the following:

$$ \mathrm{Z}\mathrm{n}{\left({\mathrm{C}}_2{\mathrm{H}}_5\right)}_2 + {\mathrm{H}}_2\mathrm{O}\to \mathrm{Z}\mathrm{n}\mathrm{O} + 2{\mathrm{C}}_2{\mathrm{H}}_6 $$

where high purity nitrogen is used as the purging gas. The warm-wall reactor is operated at the extremely low temperature of 60°C. Low-temperature growth is also a critical factor, which limits the processes of spinodal decomposition and foreign-phase formation when transition metal atoms are incorporated into the ZnO lattice [25,26]. We concentrate on the possibility of controlling the free-carrier concentration by lowering the growth temperature [22]. Here, we apply a long purging time after the H_2_O precursor of 200 s, with the purpose of making the reaction more complete. The detailed growth process is shown in Figure 2. The deposition process is set to 50 cycles, each cycle being 0.9 Å. ZnO thin films are grown on the Si substrate and the photopolymer substrate containing AgNW. The advantage of ZnO film grown on peeled off AgNW is that the ZnO film is able to completely fill the voids in the AgNW anode, also that the anode is able to contact the device all over the active area, in order to allow for efficient charge extraction/injection. The X-ray photoemission spectroscopy (XPS) is performed using a Scienta ESCA 200 spectrometer (VG Scienta, Uppsala, Sweden) in ultrahigh vacuum with a base pressure of 1 × 10^−8^ Pa. The measurement chamber is equipped with a monochromatic Al KR X-ray source to afford photons with 1,486.6 eV. The scanning electron microscopy (SEM) is performed using a field-emission SEM (JSM-6700F, JEOL, Tokyo, Japan) operated at an accelerating voltage of 15 kV. The surface morphology and root mean square (RMS) of ZnO film on Si substrate is measured with a Veeco atomic force microscope (AFM; Bruker Corporation, Billerica, MA, USA). The sheet resistance of anodes is measured by a four-probe ST-21 system (Janis, Woburn, MA, USA). A Kyoto UV-2550 (Shimadzu Scientific Instruments, Kyoto, Japan) measures the transmittance.

Hole-only devices are fabricated on the peeled off substrate with the AgNW anode and the AgNW/ZnO hybrid anode. The devices are successively deposited by employing conventional thermal evaporation at 5 × 10^−4^ Pa, without breaking the vacuum. The stack structure is as follows: a 60-nm thick 4,4′,4″-tris(N-3(3-methylphenyl)-N-phenylamino)-triphenylamine(m-MTDATA) as the hole-injection layer, a 40-nm thick N,N′-biphenyl-N,N′-bis(1-naphenyl)-[1,1′-biphenyl]-4,4′-diamine (NPB) as the hole transport layer, a 60-nm thick m-MTDATA, and a 100-nm thick Al cathode. The active area of devices is 1 cm^2^. The current density-voltage characteristics of these hole-only devices are tested using an Agilent B2902A (Agilent Technologies, Santa Clara, CA, USA) programmable voltage-current source. All the measurements are conducted in the ambient environment at room temperature.

## Results and discussion

**Figure 3:**
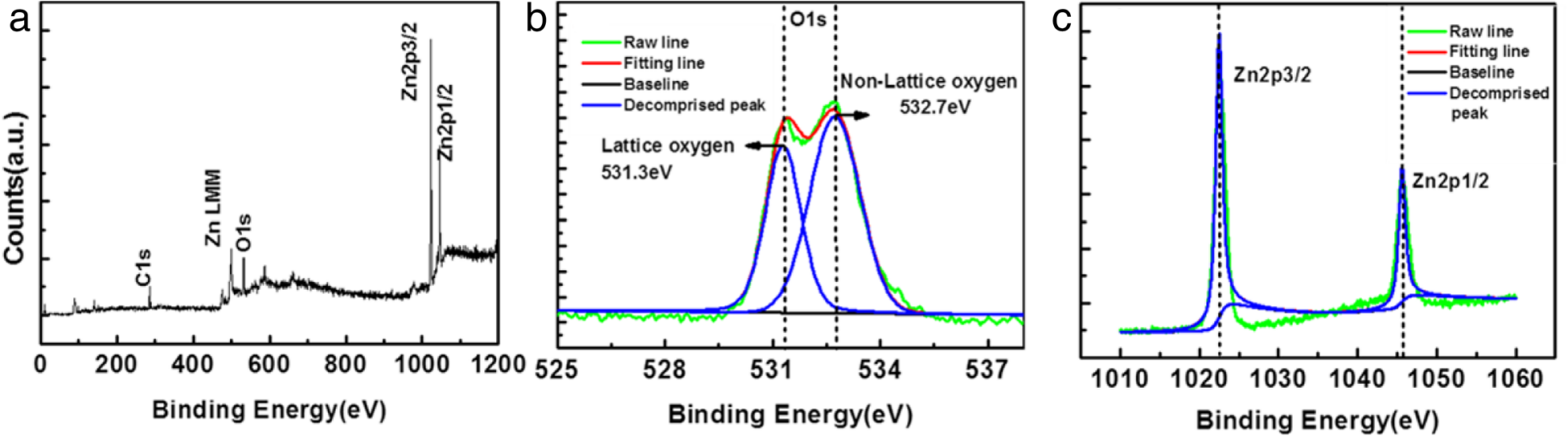
**High-resolution XPS spectra. (a)** XPS full spectrum, **(b)** the O1s, and **(c)** Zn2p of ZnO films.

The chemical bonding structure of ZnO films on a Si substrate and a photopolymer substrate are examined using XPS. Figure 3a shows the typical XPS full spectra of ZnO thin films. The C1s, O1s, Zn2p3/2, and Zn2p1/2 can be observed easily. C is caused by organic carbon pollution in the vacuum system or by pollution in the air, the latter occurring when samples are transferred to the XPS analysis chamber in the process. In order to eliminate nuclear effects, the binding energies are calibrated by taking the carbon C1s peak (284.6 eV) as a reference [27]. Figure 3b,c shows the O1s and Zn2p XPS spectra, respectively. The O1s signals shown in Figure 3b are resolved into two peaks by a Lorentzian distribution fitting. The main O1s peak component at 532.7 eV is assigned to the oxygen-deficient regions within the matrix of ZnO [28], the surface hydroxide, and the adsorbed H_2_O. The second peak at 531.3 eV is assigned to the lattice oxygen in ZnO [29]. That is to say, the intensity of the component is the measure of the amount of oxygen atoms in a fully oxidized stoichiometric surrounding. Therefore, changes in the intensity of this component may be connected in part to the variations in the concentration of oxygen vacancies. These data indicate that the ZnO film deposited at 60°C exhibit large numbers of non-lattice oxygen atoms, which may induce the formation of a non-homogeneous and non-stoichiometric multi-defect ZnO film. From Figure 3c, metallic Zn with a binding energy of 1,021.5 eV [30] does not exist, which shows that Zn is present in an oxidation state. The binding energy of Zn2p3/2 is 1,022.5 eV, which is larger than the ones corresponding to bulk ZnO and to metal Zn, indicating a dominant formation of ZnO and that most of the Zn atoms are present as Zn^2+^ within an oxygen-deficient ZnO_1 − *x*_ matrix [31]. From the Zn LMM of XPS spectra shown in Figure 3a, Auger peaks of Zn exist in ZnO in the ranges 472 to 502.5 eV; these are attributed to nearly free-state Zn, which may come from interstitial positions in the ZnO film or from nonbonding Zn [32].

**Figure 4:**
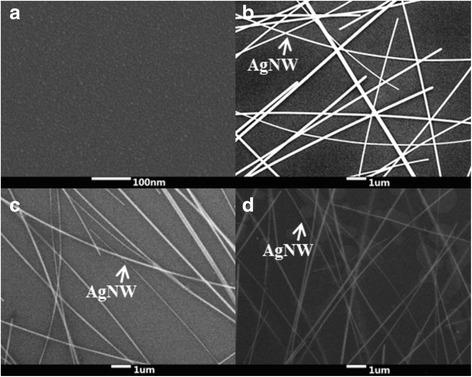
**The top surface of SEM images. (a)** ZnO grown on Si substrate; **(b)** AgNWs on Si substrate; **(c)** AgNWs after being transferred and embedded in the photopolymer substrate via spin-coating process and peel off; **(d)** ZnO-AgNWs in the photopolymer substrate.

Shown in Figure 4 are SEM top views. Each grain in the ZnO film on a Si substrate is small and has a round shape (Figure 4a). This film structure may be due to sufficient thermal decomposition of the reactants. But crystal formation requires higher energy; thus, non-homogeneous films tend to form at very low growth temperatures. The RMS surface roughness of the ZnO film on Si substrate is 1.18 nm. Figure 4b shows AgNW randomly distributed on the Si substrate. After the peel-off process, the AgNW submerges in photopolymer film and retains the original random distribution (Figure 4c). After growing the ZnO film on a AgNW electrode with photopolymer substrate, we observe several dots similar to a number of small bubbles (Figure 4d). Moreover, the more intensive the nanowire, the more dots exist. This phenomenon may be due to the slight heating (60°C) of the photopolymer substrate, which leads to a tiny photopolymer expansion, and then to a separation at the boundary between different substances.

**Figure 5:**
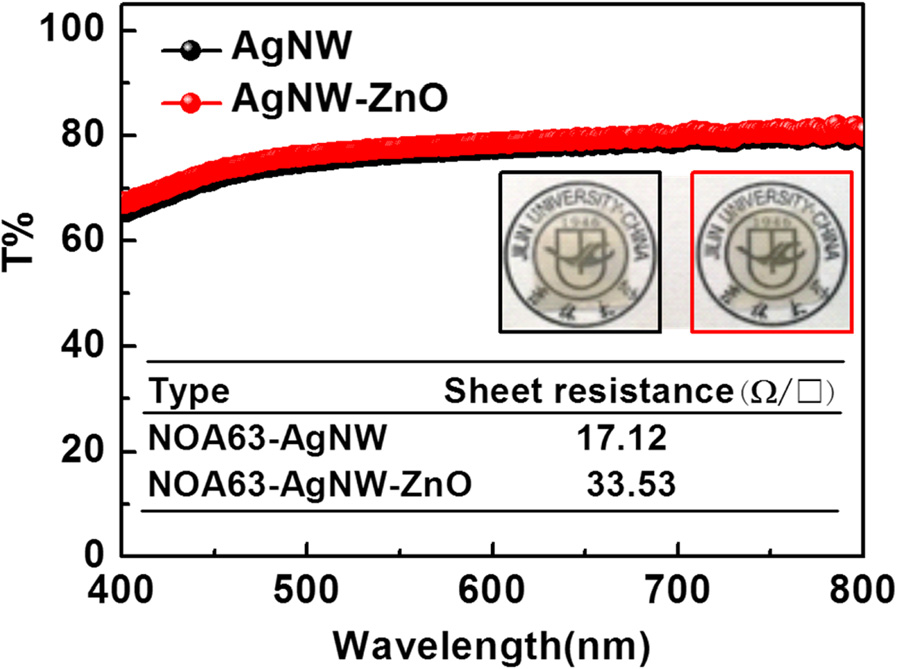
**Light transmittance of AgNW anode on photopolymer substrate before and after growing ZnO.**

**Figure 6:**
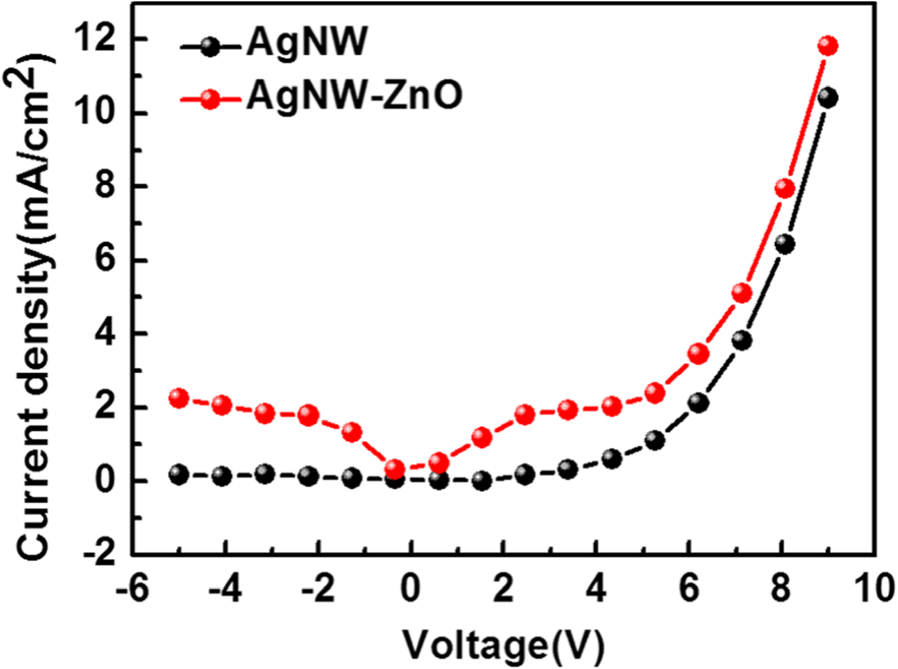
**Current-density curves of hole-only devices with AgNW and ZnO-AgNW anode on photopolymer substrate.**

The sheet resistance and the light transmittance are two important factors to evaluate electrodes used as bottom-emitting devices. The light transmittance is obtained in the wavelength range of visible light. The AgNW electrodes with photopolymer before and after ZnO film growth are measured; the results are quite close (Figure 5), but a barely imperceptible disparity exists, which is presumably due to minor differences in substrate thickness in the process of spin-coating and to instrument measurement errors. The inset of Figure 5 shows photographs of transmittance test samples. There are almost no differences between them when observed with the naked eye. The sheet resistance for two kinds of electrodes with a photopolymer substrate is also shown in the inset of Figure 5. After the growth of ZnO, the conductivity of the electrode is lower due to the ZnO film being very thin. It is speculated that if ZnO films grow too thick, both the optical and the electrical properties of AgNW/ZnO hybrid anode are getting worse. After investigating the nature of the electrodes, we utilize the AgNW/ZnO hybrid anode in a hole-only device with AgNW anode as comparison. Current density-voltage characteristics are shown in Figure 6. The hole-only device with a AgNW/ZnO hybrid anode has a higher current density than the one without ZnO at the same voltage, which indicates that ZnO is effective in enhancing the hole-injection when inserted between AgNW and NPB. Therefore, the ZnO film grown by a low temperature is significant for a modified AgNW anode.

## Conclusions

The production of a flexible electrode is the first and crucial step in the making of flexible organic electronics. In this work, ZnO films are first utilized to compose a hybrid flexible anode with peel-off Ag nanowires on a photopolymer substrate. An extremely low temperature (60°C) ALD process is used to grow ZnO films. XPS analysis shows that at a low temperature, the ALD ZnO may form a multi-defect film, which could promote conductivity. The injection of carriers has been effectively improved due to ZnO to a sufficient degree to fill the vacancy between nanowires. Low-growth temperature simultaneously guarantees the flexibility and the mechanical properties of the plastic substrate. The conductivity and the transmittance of the hybrid flexible anode are great. In summary, ZnO films grown at low-temperature ALD are important to improve the performance of the AgNW mesh electrode on polymer, which has potential as ITO substitute in improving organic optical electronics. As next steps, we will apply this hybrid electrode with photopolymer substrate obtained via a combined peel-off process to the flexible organic light-emitting or organic photovoltaic devices. We anticipate these devices to exhibit superior mechanical flexibility, transparency, and conductivity performance.

## Abbreviations

AFM: atomic force microscope
ALD: atomic layer deposition
(DEZn) [Zn(C_2_H_5_)_2_]: diethylzinc
ITO: indium tin oxide
AgNW: silver nanowire
ZnO: zinc oxide
